# Reflexões sobre a Triagem Pré-Operatória de ECG para Indivíduos Assintomáticos de Baixo Risco

**DOI:** 10.36660/abc.20240055

**Published:** 2024-07-01

**Authors:** José Nunes de Alencar

**Affiliations:** 1 Instituto Dante Pazzanese de Cardiologia São Paulo SP Brasil Instituto Dante Pazzanese de Cardiologia, São Paulo, SP – Brasil

**Keywords:** Pré-operatório, Eletrocardiograma

Li com interesse o estudo de Ramos et al. sobre a "Importância Prognóstica do Eletrocardiograma Pré-operatório em Pacientes de Baixo Risco Submetidos à Intervenção Cirúrgica sob Anestesia Geral".^1^ Os esforços dos autores para esclarecer a eficácia preditiva da eletrocardiografia (ECG) pré-operatória em uma população que parece ter um perfil de baixo risco são louváveis. No entanto, tenho preocupações quanto à metodologia empregada, que pode potencialmente impactar as conclusões do estudo.

Examinar diretamente a hipótese de que um ECG anormal pode servir como preditor de eventos é essencial. Esta análise secundária foi incorporada pelos autores dentro de um subconjunto da população que realizou ECG. Para testar de forma robusta a sua hipótese, a análise primária deveria ter sido se um ECG anormal é um preditor de risco aumentado em comparação com os resultados normais do ECG. A implementação desta metodologia produziria uma avaliação mais precisa da capacidade do ECG de prever complicações pós-operatórias.

Em segundo lugar, como um estudo randomizado e prospectivo, a adesão às diretrizes de notificação estabelecidas, como CONSORT^2^ ou SPIRIT^3^ é imperativo. Essas *checklists* defendem uma descrição metodológica transparente em relação à seleção dos pacientes, randomização, alocação e definição de desfechos primários e secundários. A falta de tais detalhes levanta preocupações sobre possíveis vieses de seleção e indicação.^4,5^ Por exemplo, selecionar inadvertidamente pacientes para eletrocardiogramas (ECGs) com base em avaliações pré-operatórias subjetivas que os colocam em um perfil de risco ligeiramente mais elevado poderia introduzir ambos os vieses.

Além disso, sem um poder de investigação predefinido e um nível alfa convencional, é um desafio determinar o tamanho da amostra necessário para obter significância estatística. A falta de definições para análise do tamanho da amostra, beta e alfa neste estudo restringe a capacidade de formulação de conclusões definitivas. Além disso, a clareza quanto ao manejo de pacientes com achados anormais no ECG é crucial para a replicação dos resultados em diferentes centros. Esta informação serve para apoiar a generalização dos resultados do estudo e orientaria a prática clínica.

Considerando os fatores acima mencionados, respeitosamente tenho opinião divergente em relação à conclusão do estudo de que o ECG pré-operatório não contribui para o prognóstico de complicações pós-operatórias em pacientes com 50 anos ou mais submetidos à cirurgia com anestesia geral sem quaisquer condições médicas importantes. Do meu ponto de vista, a metodologia utilizada na pesquisa não consegue fundamentar suficientemente esta conclusão. Uma abordagem mais apropriada envolveria uma avaliação prospectiva de pacientes com ECGs normais e anormais, calculando diferenças nas razões de risco. Em contraste, um desenho prospectivo randomizado poderia produzir resultados mais conclusivos, atribuindo aleatoriamente pacientes que apresentam eletrocardiogramas (ECGs) anormais ao tratamento padrão ou a uma terapia guiada por ECG replicável e predeterminada. A implementação de tais metodologias aumentaria a capacidade de distinguir a influência dos resultados do ECG de variáveis adicionais de confusão, proporcionando assim uma avaliação mais precisa da capacidade do ECG de prever resultados pós-operatórios.

A busca de melhorar o atendimento ao paciente por meio de práticas baseadas em evidências implica um compromisso contínuo com a educação e o progresso. É através do discurso acadêmico e de metodologias de pesquisa meticulosas que refinamos nossos protocolos clínicos para atender melhor nossos pacientes.
