# Supplementary material for: Reflexões sobre a Triagem Pré-Operatória de ECG para Indivíduos Assintomáticos de Baixo Risco
Source: Arq Bras Cardiol. 2024 Jul 1;121(6):e20240055. [Article in Portuguese] doi: 10.36660/abc.20240055 (PMC12080606; doi:10.36660/abc.20240055)
Supplement: Translation

# Translation: Reflections on ECG Preoperative Screening for Asymptomatic Low-Risk Individuals

**DOI:** 10.36660/abc.20240055i

**Authors:** José Nunes de Alencar

**Affiliations:** 1 São Paulo SP Brazil Instituto Dante Pazzanese de Cardiologia, São Paulo, SP – Brazil

**Keywords:** Preoperative, Electrocardiogram

I read with interest the study by Ramos et al. on the " Prognostic Value of Preoperative Electrocardiogram in Low-Risk Patients Undergoing Surgical Intervention and General Anesthesia".^1^ The authors’ efforts to clarify the predictive efficacy of preoperative electrocardiography (ECG) in a population that appears to have a low-risk profile are meritorious. Nonetheless, I harbor concerns regarding the employed methodology, which may potentially impact the study’s findings.

Directly examining the hypothesis that an abnormal ECG can serve as a predictor of events is essential. This secondary analysis was incorporated by the authors within a subset of the population that underwent ECG. To robustly test their hypothesis, the primary analysis should have been whether an abnormal ECG is a predictor of increased risk compared to normal ECG findings. Implementing this methodology would yield a more precise assessment of the ECG’s ability to forecast postoperative complications.

Secondly, as a randomized and prospective study, adherence to established reporting guidelines such as CONSORT^2^ or SPIRIT^3^ is imperative. These checklists advocate for a transparent methodological description concerning patient selection, randomization, allocation, and the definition of primary and secondary outcomes. The lack of such detail raises concerns about potential selection and indication biases.^4,5^ For instance, inadvertently selecting patients for electrocardiograms (ECGs) based on subjective preoperative assessments that place them at a slightly higher risk profile could introduce both of these biases.

Furthermore, without predefined research power and a conventional alpha level, it is challenging to ascertain the required sample size for statistical significance. The lack of definitions for sample size analysis, beta, and alpha in this study restricts the capacity to formulate definitive conclusions. Moreover, clarity regarding the management of patients with abnormal ECG findings is crucial for the replication of results across different centers. This information serves to support the generalizability of the study’s results and would guide clinical practice.

Considering the aforementioned factors, I respectfully hold a differing opinion regarding the study’s conclusion that preoperative ECG does not contribute to the prognosis of postoperative complications in patients aged 50 and above who are undergoing general anesthetic surgery without any major medical conditions. From my perspective, the methodology utilized in the research fails to sufficiently substantiate this conclusion. A more appropriate approach would involve a prospective evaluation of patients with abnormal and normal ECGs, calculating differences in risk ratios. In contrast, a prospective randomized design could yield more conclusive results by randomly assigning patients presenting with abnormal electrocardiograms (ECGs) to either standard care or a predetermined, replicable ECG-guided therapy. Implementing such methodologies would enhance the ability to distinguish the influence of ECG results from additional confounding variables, thus providing a more accurate assessment of the ECG’s capacity to forecast postoperative outcomes.

The pursuit of enhancing patient care via evidence-based practices entails an ongoing commitment to education and progress. It is through scholarly discourse and meticulous research methodologies that we refine our clinical protocols to serve our patients better.
