# Supplementary material for: Reflexões sobre a Triagem Pré-Operatória de ECG para Indivíduos Assintomáticos de Baixo Risco
Source: Arq Bras Cardiol. 2024 Jul 1;121(6):e20240055. [Article in Portuguese] doi: 10.36660/abc.20240055 (PMC12080606; doi:10.36660/abc.20240055)
Supplement: Related texts

# Reply: Carta-resposta

**Authors:** Lafayete Ramos, Valdir Ambrósio Moisés

Agradecemos os comentários e dúvidas levantadas pelo autor da carta "Reflexões sobre a triagem pré-operatória de ECG para indivíduos assintomáticos de baixo risco". Embora algumas questões sejam semelhantes às levantadas pelos revisores indicados pelos Arquivos Brasileiros de Cardiologia elas ajudam a nós e aos leitores a refletir sobre a melhor forma de analisar e melhorar o valor do eletrocardiograma pré-operatório.

Conforme descrito no artigo, nosso principal interesse foi investigar o valor da realização de um eletrocardiograma pré-operatório, e não seus resultados. Nossa conclusão respondeu a essa questão com base nos resultados observados e apontou para refletir se a indicação do eletrocardiograma pré-operatório deve ser baseada apenas na idade do paciente.^1^

Realizamos uma análise secundária no grupo que realizou eletrocardiograma. O subgrupo com alterações eletrocardiográficas não apresentou diferença significativa na mortalidade e morbidade em relação ao grupo com eletrocardiogramas normais. Esses achados, por serem baseados em análise secundária e com menor número de pacientes, não fizeram parte da conclusão principal do nosso estudo.^1^

O processo de randomização foi adequado; nenhuma preferência para realizar um eletrocardiograma foi feita com base na idade, complexidade e duração do procedimento cirúrgico. Conforme apontado no artigo, reconhecemos muitas limitações do estudo. Os resultados não tiveram poder suficiente para mudar o atendimento ao paciente de acordo com as diretrizes, mas sugeriram que mais estudos deveriam ser realizados na área.^2^ Estudos multicêntricos incluindo pacientes com diferentes faixas etárias, risco cirúrgico e cirurgias complexas certamente trarão mais detalhes ao assunto.

## References

[1] Ramos L, Coutinho AC, Rebelato J, Ramos MV, Elly E, Amoedo P (2024). Prognostic Value of Preoperative Electrocardiogram in Low-Risk Patients Undergoing Surgical Intervention and General Anesthesia. Arq Bras Cardiol.

[2] Fleisher LA, Fleischmann KE, Auerbach AD, Barnason SA, Beckman JA, Bozkurt B (2014). 2014 ACC/AHA Guideline on Perioperative Cardiovascular Evaluation and Management of Patients Undergoing Noncardiac Surgery: A Report of the American College of Cardiology/American Heart Association Task Force on Practice Guidelines. J Am Coll Cardiol.

# Reply

**Authors:** Lafayete Ramos, Valdir Ambrósio Moisés

We thank the comments and questions raised by the author of the letter "Reflections on ECG Preoperative Screening for Asymptomatic Low-Risk Individuals". Although some of the questions were similar to those raised by the reviewers indicated by Arquivos Brasileiros de Cardiologia they help us and the readers to reflect on the best way to analyze and improve the value of preoperative electrocardiogram.

As described in the paper, our main interest was to investigate the value of performing a preoperative electrocardiogram, not the results of them. Our conclusion responded to this question based on the results observed and pointed out to reflect if the indication of a preoperative electrocardiogram should be based only on the age of the patient.^1^

We performed a secondary analysis in the group that had an electrocardiogram performed. The subgroup with electrocardiographic alterations did not have a significant difference in mortality and morbidity compared to the group with normal electrocardiograms. These findings, as based on secondary analysis and with a lower number of patients, were not part of the main conclusion of our study.^1^

The randomization process was adequate; no preference to perform an electrocardiogram was made based on age complexity and duration of the surgical procedure. As pointed out in the paper we recognize many limitations of the study. The results did not have the power enough to change patient care according to guidelines but suggested that more studies should be performed in the field.^2^ Multicentric studies including patients with different age groups, surgical risk, and complex surgeries will certainly bring more detail to the subject.
